# Classification and characterization of class III malocclusion in Chinese individuals

**DOI:** 10.1186/s13005-016-0127-8

**Published:** 2016-11-07

**Authors:** Cai Li, Ying Cai, Sihui Chen, Fengshan Chen

**Affiliations:** Department of Orthodontics, School & Hospital of Stomatology, Shanghai Engineering Research Centre of Tooth Restoration and Regeneration, Tongji University, Shanghai, 200072 China

**Keywords:** Class III malocclusion, Mandibular prognathism, Subtypes, Multivariate, Principal component analysis, Cluster analysis

## Abstract

**Background:**

Class III malocclusion is a maxillofacial disorder that is characterised by a concave profile and can be attributed to both genetic inheritance and environmental factors. It is a clinical challenge due to our limited understanding of its aetiology. Revealing its prototypical diversity will contribute to our sequential exploration of the underlying aetiological information. The objective of this study was to characterize phenotypic variations of Class III malocclusion via a lateral cephalometric analysis in a community of Chinese individuals.

**Method:**

One-hundred-and-forty-four individuals (58 males ≥18 and 86 females ≥16) with Class III malocclusion ranging from mild to severe were enrolled in this study. Principal component analysis and cluster analysis were performed using 61 lateral cephalometric measurements.

**Results:**

Six principal components were discovered in the examined population and were responsible for 73.7 % of the variability. Four subtypes were revealed by cluster analysis. Subtype 1 included subjects with mild mandibular prognathism with a steep mandibular plane. Subjects in subtype 2 showed a combination of prognathic mandibular and retrusive maxillary with a flat or normal mandibular plane. Subtype 3 included individuals with purely severe mandibular prognathism and a normal mandibular plane. Individuals in subtype 4 had a mild maxillary deficiency and severe mandibular prognathism with the lowest mandibular plane angle.

**Conclusion:**

The six principal components extracted among the 61 variables improve our knowledge of lateral cephalometric analysis for diagnoses. We successfully identified four Class III malocclusion subtypes, indicating that cluster analysis could supplement the classification of Class III malocclusion among a Chinese population and may assist in our on-going genetic study.

**Electronic supplementary material:**

The online version of this article (doi:10.1186/s13005-016-0127-8) contains supplementary material, which is available to authorized users.

## Background

Class III malocclusion has long been considered a complicated maxillofacial disorder that is characterised by a concave profile, which may exhibit mandibular protrusion, maxillary retrusion or a combination of both [1] as well as possible anatomic heterogeneity of this malocclusion. The prevalence of Class III malocclusion varies greatly both among and within populations, and the highest prevalence of 15.8 % has been observed in Southeast Asian populations in previous studies [2]. In recent years, it has been widely accepted that both genetic inheritance and environmental factors contribute to Class III malocclusion [3, 4], and diversity loci and suspicious genes associated with Class III malocclusion have been identified using linkage analysis and association studies [4–10]. Although informative, the previous genetic studies have limitations, including modest sample sizes, the exclusion of environmental factors, the lack of a systematic estimation of genetic variants associated with the disease, and perhaps more importantly, limited phenotypes that cannot capture the complexities of Class III malocclusion [11]. Owing to the limited knowledge of the underlying aetiologies of this condition, it is still a challenge for dentists to diagnose and treat Class III malocclusion [12]. Distinguishing phenotypes that are related to different expressions of a genotype is an essential step in establishing the genetic contribution to Class III malocclusion.

Lateral cephalometric radiographs provide rich phenotypic data, which provide information about the cranial, facial bony and soft tissue structures. Cephalometric analysis is an economic and convenient accessory examination and plays a predominant role in approaching the definition of phenotypes among and within the Class III population [13, 14]. Recently, multivariate analyses such as discriminant analyses, principal component analyses (PCA) and cluster analyses have been used to distinguish the phenotypic variations of Class III malocclusion in several studies [11, 14–16]. PCA is a powerful method that is used to provide an overview of complex multivariate data [17]. In contrast, cluster analysis complements PCA organized variables to select homogeneous information such that the underlying phenotype may be identified. This method has also been applied to determine the subtypes of other diseases [18–20].

A large sample of patients with Class III malocclusion from the University of North Carolina was studied by Bui et al., including a wide age range from 5.9 to 56.3 years and racial diversity [14]. In this study, five clusters were identified to represent distinct subtypes via PCA and cluster analyses. Recently, Moreno Uribe et al. characterized Class III malocclusion phenotypes by using the same method with 63 cephalometric measures derived from 292 Caucasian adults. The PCA reduced 63 cephalometric variables into six principal components that explained 81 % of the variability within the samples, and the cluster analysis classified the individuals into five distinct subtypes, which differed from the findings of previous research [11].

Although a few previous studies have contributed to the characterization of Class III malocclusion, there is still uncertainty about whether Class III phenotypic classifications can be generalizable to other samples and populations. We may identify different phenotypic subgroups specific to the Chinese population. Our group has been engaged in genetic studies of Class III malocclusion and has obtained important findings [21–23]. In this study, we aimed to identify additional phenotypic variation within a large group of Chinese samples using methods similar to those of Moreno Uribe. These findings will facilitate clinical diagnoses and will enhance future genetic studies.

## Methods

### Study samples

We enrolled 144 subjects (58 males ≥18 and 86 females ≥16), with a clinical diagnosis of Class III malocclusion who were seeking orthodontic treatment at the Affiliated Stomatology Hospital of Tongji University from January 2014 to September 2015. The subjects ranged in age from 16 to 35 years, with a mean age of approximately 23 (22.61 ± 4.58) years. All participants were of Han Chinese ancestry and their conditions ranged from mild to severe phenotypes, and the patients all met at least two of the eligibility criteria (Table 1), including an ANB angle (Point A-Nasion-Point B) of the centric jaw relationship < 0.0°, an anterior crossbite, and a Wits appraisal greater than −2.0 mm [3, 4, 24]. Participants who had previous orthodontic treatments, congenital abnormalities (*e.g.,* cleft lip and palate), severe facial trauma, or general physical disease (*e.g.,* endocrine diseases) were excluded.

**Table 1:** Characteristics of the study group

	Inclusion Criteria	Exclusion Criteria
1	ANB ≤ 0°	History of orthodontic treatments
2	Overjet ≤ 0 at least edge-to-edge or anterior crossbite	Congenital abnormalities (e.g., cleft lip and palatee)
3	Wits ≤ −2°	Severe facial trauma
4		General physical disease (e.g., endocrine diseases)

### Cephalometric analysis

All the lateral cephalograms involved in our study were digital films. The exposures were made by a standardized technique with the patients’ jaws in centric occlusion with an equipment of dental X-ray (Veraviewepocs X550, Kyoto, Japan). Captured images were saved as JPG files. The obtained digital radiographs were then standardized with a 10-mm ruler and imported into the NemoCeph NX software (version 6.0, Nemotec, Madrid, Spain). Cephalometric tracing and measurement were performed using the analysis software with a computer by an experienced orthodontist. Sixty-one cephalometric parameters digitized with 20 skeletal landmarks and 10 soft tissue landmarks were selected, which represented comprehensive craniofacial information, including information about the skeletal structure, teeth, soft tissue and their relationships to each other (Table 2). An additional file shows the data of the cephalometric analysis in more detail (see Additional file 1). A sample of 15 random lateral cephalograms were traced twice at least 2 weeks apart. The reliability of the landmark location (intra-examiner agreement) was assessed using intra-class correlation methods (ICC) [25]. The result showed that the intra-examiner reliability ranged from ICC = 85.21 % to ICC = 99.99 %, which is generally acceptable.

**Table 2:** Cephamotric variables

**Cranial Base**	Condylion to Gnathion (Co-Gn)(mm)	IMPA (L1-MP) (°)
Saddle/Sella Angle (SN-Ar) (°)	**Intermaxillary**	L1-NB (°)
Anterior Cranial Base (SN) (mm)	Midface Length (Co-A) (mm)	L1-NB (mm)
Posterior Cranial Base (S-Ar) (mm)	ANB (°)	L1 Protrusion (L1-APg) (°)
**Maxilla**	Facial Plane to AB (NP-AB) (°)	L1 Protrusion (L1-APg) (mm)
SNA (°)	Post-Ant Face Height (S-Go/N-Me) (%)	FMIA (L1-FH) (°)
Convexity (NA-APg) (°)	Y-axis (N-S-Gn) (°)	Interincisal Angle (U1-L1) (°)
N to A through the Horizontal Plane (mm)	Maxillary-Mandibular Difference (mm)	UADH (U1-PP) (mm)
Na _|_ to A point (mm)	Wits Appraisal (AO-BO) (mm)	LADH (L1-MP) (mm)
Maxilla Length (Co-ANS) (mm)	Anterior Face Height (N-Me) (mm)	UPDH (U6-PP) (mm)
**Mandible**	Upper Face Height (N-ANS) (mm)	LPDH (L6 - MP) (mm)
SNB (°)	Inferior Facial Height (mm)	Overjet (mm)
Facial Angle (FH-NPg) (°)	Nasal Height (N-ANS/N-Me) (%)	Overbite (mm)
Gonial Angle (Ar-Go-Me)(°)	MP-SN (°)	**Soft Tissue**
Ramus Height (Ar-Go) (mm)	FMA (FH-MP) (°)	Upper Lip to E-Plane (mm)
Facial Taper (N-Gn-Go) (°)	GoGn-SN (°)	Lower Lip to E-Plane (mm)
Articular Angle (S-Ar-Go) (°)	Occlusal plane To SN (°)	Upper lip length (Sn-ULI)
N to B through Horizontal Plane (mm)	Occlusal Plane to FH (OP-PoOr) (°)	Lower lip length (LLS-Me')
N to Pg through Horizontal Plane (mm)	**Dental**	Facial angle (G'-Sn-Pog') (°)
Pg to Na Perpendicular (mm)	U1-SN (°)	Upper lip anterior (ULA-TVL) (°)
Mandibular Unit Length (Co-Gn) (mm)	U1-NA (°)	Lower lip anterior (LLA - TVL) (°)
Pg - NB (mm)	U1-NA (mm)	
Posterior Facial Height (Co-Go) (mm)	U1-FH (°)	

Boldface indicates six categories of the sixty-one cephalometric parameters

### Statistical analysis

All measured values were adjusted with multiple linear regression to assess the possible effects of age and gender and eliminate the interaction of age and gender. It was necessary to systematically search for factors that impacted the variables and to group these factors into homogeneous categories. Principal component analysis (PCA) was performed, and 61 principal component scores were then calculated one by one to eliminate interactions between variables. Components with a cumulative variance > 70 % were used in the following cluster analysis. Partitioning cluster analysis (CA) based on principal components (PCs) was applied to construct a hierarchical structure in all of the Class III malocclusion individuals. We performed CA by the k-means method, which sorted participants into groups by maximizing differences and minimizing differences [26, 27]. The clustering algorithm was performed separately for a range of 3 to 6 clusters. A three-dimensional plot was produced using the R statistical program to implement the visualization of the cluster analysis results. The representative subject that was closest to the mean values of the cluster was chosen as the template. One-way analysis of variance (ANOVA) and the Wilcoxon signed rank test were performed to compare the commonly used variables among each cluster, with the aim of identifying major differences across groups. In this study, IBM SPSS 22.0 was used for all analyses, and the significant difference level was set as *p* < 0.05.

## Results

**Fig. 1:**
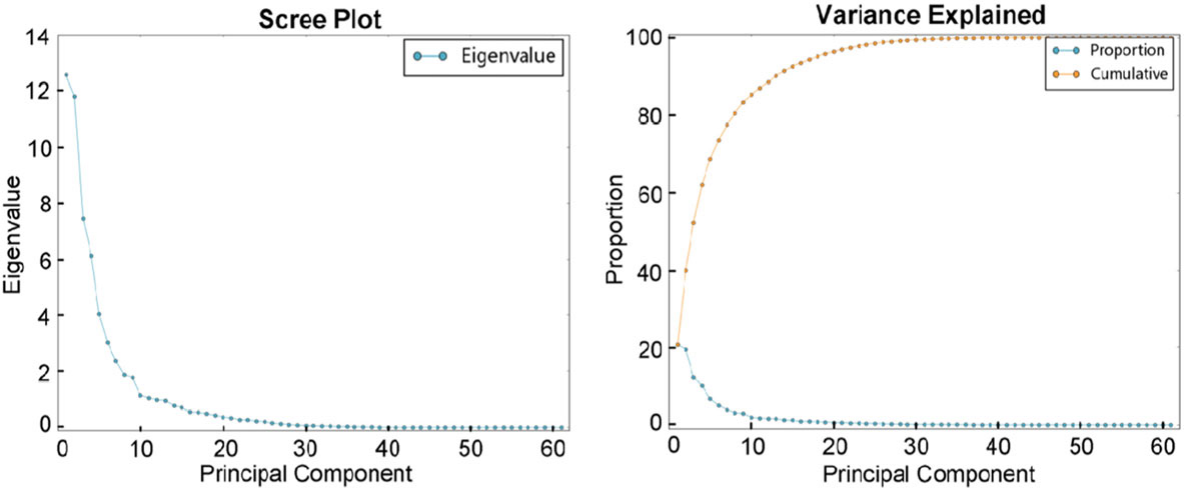
Principal Component Analyses. Six principal components accounted for 73.7 % of the variation

PCA transformed the 61 selected variables into 61 independent components. The first 6 PCs contributed significantly to representing the relationship of the 61 variables chosen for cluster analysis, which accounted for 73.7 % of all variation (Fig. 1). The first principal component (PC 1) that contributed most of the variation (20.59 %) mainly consisted of vertical length measurements. The second principal component (PC 2), which explained 19.34 % of the variation, mainly referred to the vertical and sagittal positions of the mandible in relation to the cranial base. The third principal component (PC 3) represented the protrusion and inclination of the lower incisor and explained 12.17 % of the variation. Principal component 5 (PC 5) consisted mainly of parameters for the upper incisor and accounted for only 6.60 % of the variation. Components 4 and 6 were highly correlated with the Na _|_ to A point, APDI (NP-FH), Ao-Bo (Wits), overbite (mm), and the articular angle, which cannot be easily summarized anatomically. Table 3 summarizes the correlations of the identified principal components and the variables making the greatest contributions. An additional file shows the results of the PCA in more detail (see Additional file 2).

**Table 3:** Summary of the principal components analysis

Principal component	1	2	3	4	5	6
Variance explained^a^	0.20586	0.19340	0.12168	0.10028	0.06596	0.04932
Cumulative variance^b^		0.39926	0.52094	0.62122	0.68718	0.73650
Variables^c^	Posterior Facial Height (Co-Go) (mm)	GoGn-SN (°)	LI-NB (°)	Na _|_ to A point (mm)	UI-SN (°)	Wits Appraisal (AO-BO) (mm)
	Upper Face Height (N-ANS) (mm)	MP-SN (°)	LI-NB (mm)	Pg to Na Perpendicular (mm)	U1-FH (°)	Convexity (NA-APg) (°)
	Midface Length (Co-A) (mm)	Facial Taper (N-Gn-Go) (°)	L1 Protrusion (L1-APg) (mm)	Facial Angle (FH-NPg) (°)	U1-NA (°)	Overbite (mm)
	LPDH (L6-MP) (mm)	Gonial Angle (Ar-Go-Me) (°)	L1 Protrusion (L1-APg) (°)	N to B through the horizontal plane (mm)	U1-NA (mm)	Facial angle (G' - Sn - Pog') (°)
	LADH (LI-MP) (mm)	Post-Ant Face Height (S-Go/N-Me) (%)	FMIA (L1-FH) (°)	N to A through the horizontal plane (mm)	Occlusal plane to FH (OP-PoOr) (°)	Articular Angle (S-Ar-Go) (°)

^a^represents the variance explained by each principal component in PCA

^b^shows the cumulative variance explained by each added PC sequentially

^c^displays the variables contributing the most in each PC

**Fig. 2:**
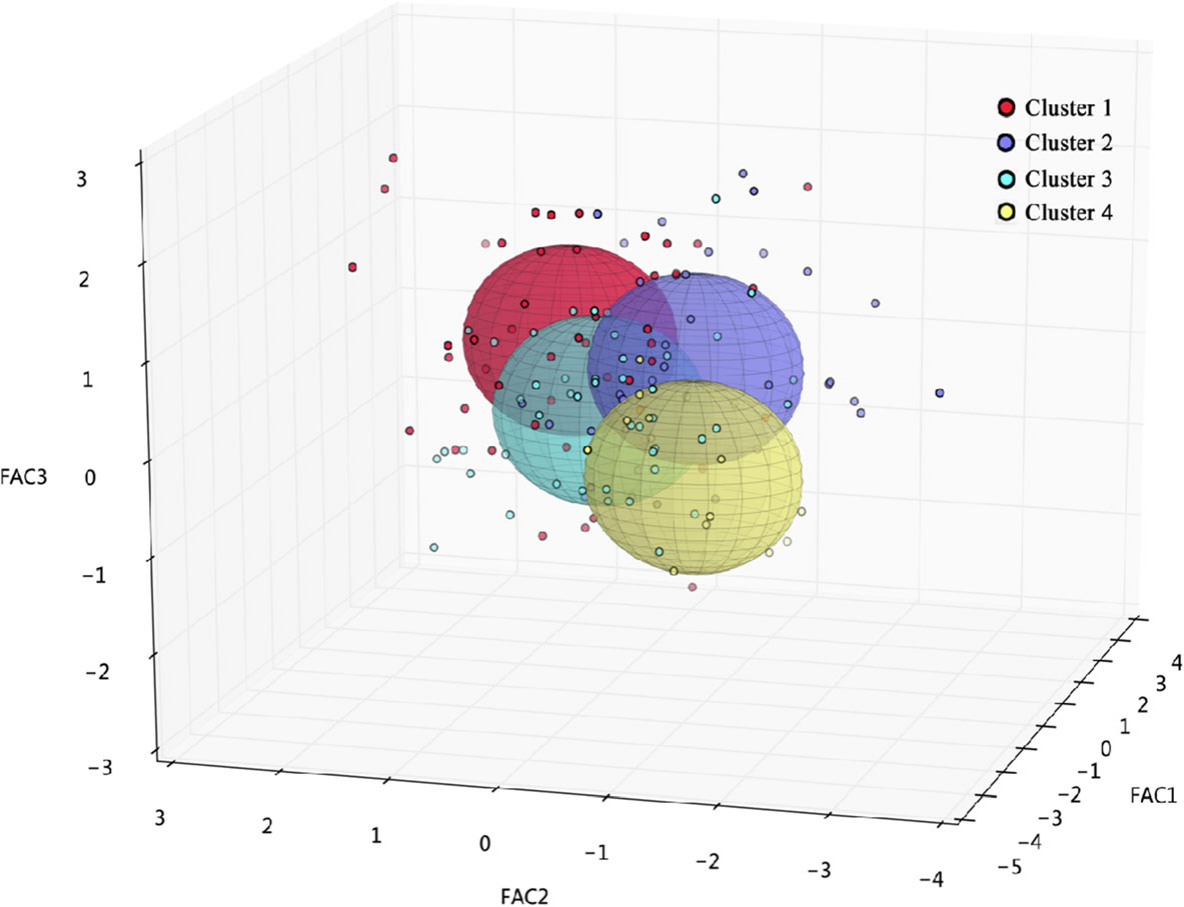
Cluster analysis results of Class III malocclusion. A 3-D spherical image representing the four identified clusters. Each cluster is traced by a unique colour

**Fig. 3:**
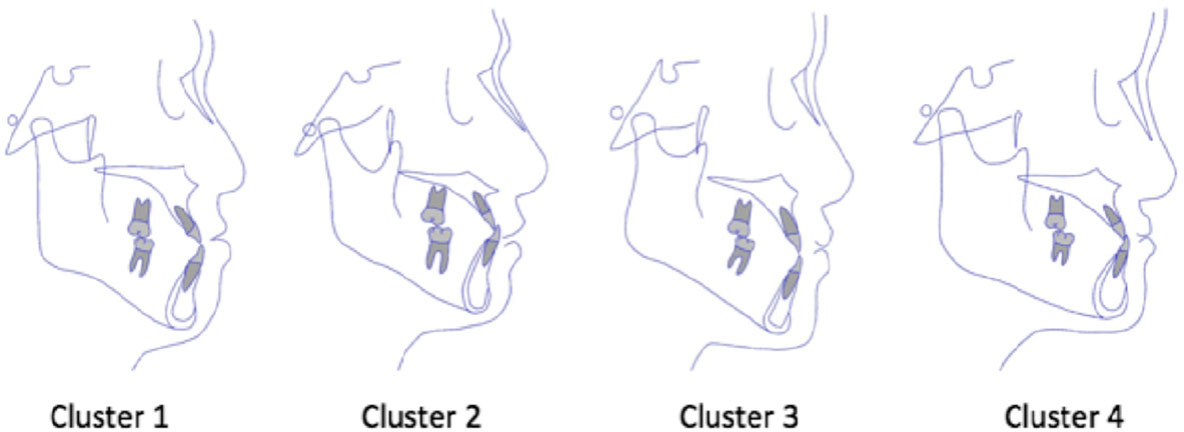
Cluster templates. The cephalometric trace of the templates in each cluster as described in the results

This group of 144 individuals with class III malocclusion were subjected to cluster analysis (CA) and were classified into 4 groups (Fig. 2; Table 4), which are both clinically meaningful and statistically acceptable based on the value of K-means in the classifier: Cluster 1 (*n* = 48) was a vertical type of Class III malocclusion that showed mild mandibular prognathism with a steep mandibular plane, and a labial inclination of the upper incisors. This group contained the largest number of observations. Cluster 2 (*n* = 38) represented individuals with moderate skeletal Class III malocclusion with a combination of a prognathic mandibular and a retrusive maxilla and a flat or normal mandibular plane. Cluster 3 (*n* = 46) was centrally located, and the subjects with this type had severe mandibular prognathism, a normal mandibular plane, and the most serious lingual inclination of the lower incisors. Subjects in Cluster 4 had the most severe phenotype of skeletal Class III malocclusion, exhibiting maxillary deficiency and severe mandibular prognathism with the lowest mandibular plane angle and an obvious labial inclination of the upper incisors. Cluster 4 also had the fewest observations (*n* = 12). Figure 3 displays templates of each cluster. Table 5 presents the descriptive statistics of each subtype, including the means and standard deviations for the variables used in each cluster, and the p-values for the significance level of each cluster are shown in Table 6. The most significant difference was observed in the FH-MP variable among the four clusters, but no evident difference was found between each cluster for the SNB and Wits variables. An additional file shows the results of the CA in more detail (see Additional file 3).

**Table 4:** Summary of the clusters

Cluster	Frequency (%)	Standard Deviation^a^	Nearest Clusters	Distance
1	48(33.3 %)	0.63	3	1.89
2	38(26.4 %)	0.58	3	2.05
3	46(31.9 %)	0.46	1	1.89
4	12(8.3 %)	0.67	3	2.54
Total	144			

^a^indicates the average distance between subjects within each cluster

**Table 5:** Means and standard deviations of variables in each cluster

Variables	Cluster 1	Cluster 2	Cluster 3	Cluster 4
Frequency	48	38	46	12
Proportions	(33.33 %)	(26.39 %)	(31.94 %)	(8.33 %)
Age	21.29 ± 3.85	24.21 ± 5.03	21.87 ± 4.38	25.67 ± 4.10
Sex(m/f)	22/26	18/20	15/31	3/9
SNA(°)	81.50 ± 3.57	80.08 ± 2.78	82.95 ± 3.28	79.95 ± 3.72
SNB(°)	83.70 ± 4.06	83.08 ± 3.12	84.40 ± 3.83	85.01 ± 4.38
ANB(°)	−2.20 ± 2.07	−3.02 ± 1.98	−1.46 ± 1.72	−5.06 ± 1.77
Wits(°)	−7.56 ± 4.02	−7.48 ± 3.66	−7.77 ± 3.40	−5.98 ± 3.42
FH-MP(°)	30.95 ± 5.49	24.16 ± 5.32	26.82 ± 5.01	18.23 ± 3.98
UI-SN (°)	118.25 ± 6.07	108.85 ± 5.94	112. 80 ± 4.85	119.69 ± 8.29
LI-MP (°)	84.00 ± 8.35	88.47 ± 8.31	82.12 ± 7.96	88.19 ± 3.90

**Table 6:** P-values of two-way cluster comparisons

	1–2	1–3	1–4	2–3	2–4	3–4
SNA(°)	0.048^*^	0.035^*^	0.147	<0.001^*^	0.908	0.006^*^
SNB(°)	0.450	0.370	0.287	0.112	0.126	0.623
ANB (°)	0.053	0.061	<0.001^*^	<0.001^*^	0.002^*^	<0.001^*^
Wits (°)	0.926	0.777	0.186	0.721	0.219	0.135
FH-MP(°)	<0.001^*^	<0.001^*^	<0.001^*^	0.021^*^	0.001^*^	<0.001^*^
UI-SN (°)	<0.001^*^	<0.001^*^	0.450	0.003^*^	<0.001^*^	<0.001^*^
LI-MP (°)	0.011^*^	0.255	0.105	<0.001^*^	0.916	0.020^*^

^*^ *P*-values < 0.05

## Discussion

By the end of the nineteenth century, Angle had first classified malocclusions into three groups (Class I, Class II and Class III) based on the relationship of the first molars; shortly thereafter, it was recognized that this classification could not capture the breadth of clinical characteristics. Gradually, Class III malocclusion was extended to refer to the skeletal jaw relationship in a mesial position of the mandible to the maxilla [2]. Class III malocclusion was a mixture of various patterns of maxillofacial deformity rather than a homogenous group. Organization of the phenotypic heterogeneity into its underlying hierarchical structure is of great necessity and may contribute to both etiological and therapeutic studies. In this study, principal component analysis and cluster analysis were performed using luxury lateral cephalometric measurements, which is a method that is frequently applied in classifications, especially when there are numerous variables. Sixty-one morphological features were included in the study, which may permit a comprehensive evaluation.

In the principal component analysis, six PCs were identified from the 61 variables among the 144 participants, which were responsible for 73.7 % of the variation. Additionally, the variables in the first three PCs explained more than half (52.09 %) of the variation. PC 1 and PC 2 consisted mainly of vertical and sagittal parameters that defined the relationship of the mandible to the cranial base, whereas PC 3 characterized the protrusion and inclination of the lower incisors. This result almost corresponds to the earlier studies by Moreno Uribe and Bui [11, 14]. Interestingly, the ANB angle (Point A-Nasion-Point B) and the SNA and SNB angles were not captured in our study, whereas these variables existed for PC 1 in the PCA performed by Moreno Uribe and Bui. Perhaps the individuals who were recruited to our study had only mild and moderate cases of class III malocclusion, and the number of severe patients may have been relatively small. Moreover, some parameters, such as facial taper, the articular angle, and the facial angle, acted as vital parts of the principal components, thus indicating their important role as measurements of Class III malocclusion. PCA was applied to reduce the interaction among the variables on which CA was performed to eliminate noisy variables that may corrupt the cluster structure [28].

Although the existence of Class III malocclusion subtypes is recognized by researchers, a few subgroups were identified among Class III malocclusion patients, three of which are defined by a long face, an average face or postural Class III [16]. Because there is a variation in the determination of the number of clusters, subjective factors could not be completely avoided in the CA. In previous studies, the patterns of five and seven clusters were proposed following a cluster analysis of more detailed cephalometric measurements [11, 14, 15]. In this research, the clustering algorithm was performed separately for a range of 3 to 6 clusters. According to our results, the model with three clusters was too simple to summarize the clinical variations, whereas in the models that included five or six clusters, one of the clusters contained fewer than five cases. Thus, we determined the existence of four subtypes of Chinese individuals based on CA.

Compared with the previous studies conducted by Moreno Uribe and Bui, who captured 5 clusters by CA [11], the subtype of severe Class III malocclusion with a retrusive maxilla and a high angle was not observed in our study, which may have been due to the moderate sample size. In addition, the proportion of people in each subtype differed from their results. A study related to the dento-facial profile of the Polish population found specific characteristics compared with other European populations. This may indicate that nationality should be considered when diagnosing facial structures [29]. Although the previous studies helped us expand the threshold of the types of Class III malocclusion, a systemic analysis to validate a practical classification system is necessary and should be the first step toward a comprehensive and accurate understanding of heterogeneity owing to ethnicity and large samples. The subjects of this study were Chinese adults and post-pubertal individuals who were not included in previous studies, which is a supplement to further systematic reviews.

In this study, a description of phenotypes based on a Chinese population was more detailed than in previous studies and was achieved by comparing the means of some commonly used measurements, such as the SNA angle, SNB angle, ANB angle, FH-MP angle, Wits and incisor angulation. The FH-MP angle rather than the ANB or SNB was the dominant classifier. Depending on the results of the PCA, this may suggest that the growth patterns rather than severity are involved in genotypes. Meanwhile, the lingual inclination of incisors in severe Class III malocclusion was significantly different from that of mild cases, which reminded orthodontists of the limitations of inclining incisors during the camouflage treatment [30]. In addition, differences in the Wits appraisal, which is usually measured to predict whether the Class III patient is a poor or good grower, were also observed to be less significant when compared among clusters, indicating that Wits might be a confusing and ambiguous measurement for assessing Class III conditions.

When discussing these results, we must consider some limitations. It is regrettable that there was a lack of family history data, which are important for the assessment of disease progression and may be closely linked to certain subtypes. In Class III malocclusion patients, diagnosis and treatment are not only influenced by severity, the jaw discrepancy, the incisor inclination, and the mandibular plane but also by factors such as age and family history, which were not included in this study. Compared with the research conducted by Moreno Uribe a few years ago in Caucasian Class III samples [11], there is a necessity to enlarge our working sample size to approach a more clinically impeccable classification system. As auxiliary examinations have increased in recent years, Cone beam CT (CBCT) identifies three-dimensional landmarks of the maxillofacial region [31, 32]. A larger sample size, including informative data extracted from CBCT, will assist in the development of a more sophisticated classification system and a more accurate understanding of the genetic aetiology. As stated previously, the cephalograms were taken in centric occlusion in this study. We found 3 patients who have an antero-posterior shift in centric relation and centric occlusion. In these 3 cases, the cephalograms were taken in both centric occlusion and centric relation. Comparing the results of the cepholametric measurement, we found little difference between centric occlusion and centric relation of the three cases. That wouldn’t cause significant influence on the final results. While among a larger sample size, it is appropriate that the cephalograms be taken in centric relation in those cases during the further studies.

Our ultimate goal is to describe the variants of Class III malocclusion and identify the genetic basis of the disease. The replication of genetic variant studies in Class III malocclusion and many other complex diseases is rare [33]. For example, what we previously identified in a large Class III malocclusion pedigree was inconsistent with the loci identified in other studies [4–10]. Considering all of these limitations, disease heterogeneity may be a difficult factor. A novel taxonomy via cluster analysis might facilitate genetic research. Additionally, clinical relevance should be investigated across subgroups in therapy after the completion of approximately 2-year-long treatment procedures in longitudinal studies. The integration of therapies related to craniofacial phenotypes would eventually lead to improved and distinct treatment schedules.

## Conclusions

Cluster analysis produced four clusters of Class III malocclusion, which represented characteristics of maxillary or mandibular discrepancy, corresponding to short or long faces, the inclination of the incisors and severity. With PCA, six PCs were extracted from the 61 variables among 144 participants, which were responsible for 73.7 % of the variation. Our study provided much more detailed information relative to previous studies by applying ANOVA and the Wilcoxon signed rank test to the variables in each cluster.

## Additional files

Additional file 1: Cephalometric Analysis. This file contains general data (number, age and gender) and the 61 cephalometric parameters of 144 subjects. (XLSX 103 kb)
Additional file 2: Principal Component 1 and Principal Component 2. Principal Component 1 displays the variables of each principal component and Principal Component 2 shows the variance explained by each principal component. (XLS 39 kb)
Additional file 3: Results of Cluster Analysis. This file shows the original results of the cluster analysis. (XLSX 55 kb)

## Abbreviation

CA: Cluster analysis
PC: Principal component
PCA: Principal component analysis
